# Neuroprotective Effects of Ginsenosides against Cerebral Ischemia

**DOI:** 10.3390/molecules24061102

**Published:** 2019-03-20

**Authors:** Zhekang Cheng, Meng Zhang, Chengli Ling, Ying Zhu, Hongwei Ren, Chao Hong, Jing Qin, Tongxiang Liu, Jianxin Wang

**Affiliations:** 1 School of Pharmacy, Minzu University of China & Key Laboratory of Ethnomedicine, Ministry of Education, Beijing 100081, China; azhekang@163.com; 2 Department of Pharmaceutics, School of Pharmacy, Fudan University & Key Laboratory of Smart Drug Delivery, Ministry of Education, Shanghai 201203, China; renhongwei0529@sina.com (H.R.); cpuhongchao@126.com (C.H.); qinjingyx@sina.com (J.Q.); 3 School of Pharmacy, Shanghai University of Traditional Chinese Medicine, Shanghai 201203, China; wlstzm561@126.com; 4 School of Pharmacy, Chengdu University of Traditional Chinese Medicine, Chengdu 610072, China; l289632020@sina.com; 5 Institute of Tropical Medicine, Guangzhou University of Chinese Medicine, Guangzhou 510405, Guangdong, China; zhuying_rain_xyz@163.com; 6 Institute of Integrative Medicine, Fudan University, Shanghai 201203, China

**Keywords:** ginsenosides, ischemia reperfusion, neuroprotective effect, inflammation, TLR4/MyD88, SIRT1

## Abstract

Ginseng has been used worldwide as traditional medicine for thousands of years, and ginsenosides have been proved to be the main active components for their various pharmacological activities. Based on their structures, ginsenosides can be divided into ginseng diol-type A and ginseng triol-type B with different pharmacological effects. In this study, six ginsenosides, namely ginsenoside Rb1, Rh2, Rg3, Rg5 as diol-type ginseng saponins, and Rg1 and Re as triol-type ginseng saponins, which were reported to be effective for ischemia-reperfusion (I/R) treatment, were chosen to compare their protective effects on cerebral I/R injury, and their mechanisms were studied by in vitro and in vivo experiments. It was found that all ginsenosides could reduce reactive oxygen species (ROS), inhibit apoptosis and increase mitochondrial membrane potential in cobalt chloride-induced (CoCl_2_-induced) PC12 cells injury model, and they could reduce cerebral infarction volume, brain neurological dysfunction of I/R rats in vivo. The results of immunohistochemistry and western blot showed that the expression of Toll-like receptor 4 (TLR4), myeloid differentiation factor 88 (MyD88), silencing information regulator (SIRT1) and nuclear transcription factor P65 (NF-κB) in hippocampal CA1 region of some ginsenoside groups were also reduced. In general, the effect on cerebral ischemia of Rb1 and Rg3 was significantly improved compared with the control group, and was the strongest among all the ginsenosides. The effect on SIRT1 activation of ginsenoside Rb1 and the inhibition effect of TLR4/MyD88 protein expression of ginsenoside Rb1 and Rg3 were significantly stronger than that of other groups. The results indicated that ginsenoside Rg1, Rb1, Rh2, Rg3, Rg5 and Re were effective in protecting the brain against ischemic injury, and ginsenoside Rb1 and Rg3 have the strongest therapeutic activities in all the tested ginsenosides. Their neuroprotective mechanism is associated with TLR4/MyD88 and SIRT1 activation signaling pathways, and they can reduce cerebral ischemic injury by inhibiting NF-κB transcriptional activity and the expression of proinflammatory cytokines, including interleukin-1β (IL-1β), tumor necrosis factor-α (TNF-α) and interleukin-6 (IL-6).

## 1. Introduction

Cerebral ischemia is a common refractory brain disease that seriously endangers human health. There are 15 million new stroke patients worldwide each year, and six million people die from the disease, among which ischemic stroke accounts for 80% [1,2]. Cerebral ischemia-reperfusion (I/R) injury is the damage caused by the recovery of blood supply after a certain period of brain tissue ischemia. After reperfusion, the dysfunction and structural damage of the brain tissue will be aggravated. A series of pathophysiological changes, including ion balance disorders, energy metabolism disorders, inflammation, glutamate excitotoxicity, oxidative stress and apoptosis, will occur after the reperfusion [3,4,5]. Inflammation plays an important role in the process of ischemic stroke. In the initial stage of inflammation, microglia and astrocytes are activated in the cerebral ischemia, and cytokines and chemokines cross the activated vascular wall to reach the penumbra [6,7]. In the later stage of inflammation, there are a large number of inflammatory factors in the penumbra. It has been reported that IL-1β, IL-6 and TNF-α are the primary inflammatory factors involved in ischemic brain damage. They play a pivotal role in the inflammatory processes that increase leukocyte adherence to blood vessels and their subsequent infiltration into the brain, meanwhile, they can damage the endothelium and lead to ischemic neural damage [8]. It is important to apply thrombolytic therapy and inhibit the inflammation after reperfusion injury to treat ischemic cerebrovascular disease. Currently, thrombolytic or neuroprotective agents are commonly used to restore the neurological function of the defect in clinic. However, the therapeutic efficacy of the available drugs is still limited.

I/R is usually accompanied by a neuroinflammatory reaction leading to excessive release of inflammatory chemokines, cytokines and adhesion molecules, which will further aggravate brain damage [9]. Toll-like receptor 4 (TLR4) is a membrane protein receptor which expresses in nerve cells and is responsible for the inflammatory response. Overexpressed TLR4 typically activates the TLR4 adaptor protein myeloid differentiation primary response gene 88 (MyD88). TLR4/MyD88 signaling will further stimulate complexes including interleukin-1 receptor-associated kinase 1 (IRAK1) and TNF receptor-associated factor 6 (TRAF6), and increases the level of nuclear factor kappa B (NF-κB) transcriptional activity and inflammatory factors in the ischemic brain. Silent information regulator 1 (SIRT1) is a histone deacetylase whose activity is dependent on nicotinamide adenine dinucleotide (NAD) and can inhibit p53 and NF-κB-induced inflammatory pathways by deacetylation [10,11]. The signaling pathway activated by TLR4/MyD88 and SIRT1 in the penumbra is the main cause of neuroinflammation in I/R, and thus a therapeutic target for neuroprotective agents.

**Figure 1:**
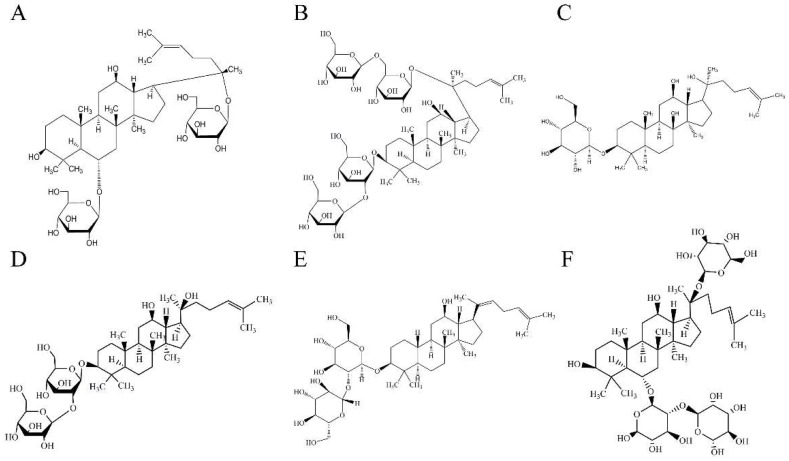
The chemical structures of ginsenosides. Ginsenoside Rg1 (**A**), ginsenoside Rb1 (**B**), ginsenoside Rh2 (**C**), ginsenoside Rg3 (**D**), ginsenoside Rg5 (**E**) and ginsenoside Re (**F**).

*Panax ginseng* Meyer belongs to the Araliaceae and is a perennial herb. Ginseng has been used as a herbal drug for thousands of years, and is widely used in the treatment of cerebrovascular diseases in Asian countries. Ginsenosides are found to be the main active ingredients of ginseng and a variety of ginsenosides could protect brain tissue from I/R damage [12,13,14,15,16]. However, to our knowledge, it is unclear which kind of ginsenoside has the strongest I/R protective effect and has potential to be an effective drug [17]. In order to compare the efficacy of various ginsenosides on I/R, six kinds of ginsenosides (Figure 1) which are commonly used for the treatment of I/R are selected based on their structure and pharmacological activities. Their effect on the apoptosis, mitochondrial transmembrane potential (MMP) and the level of reactive oxygen species (ROS) of neuron cells was studied in vitro, and their efficacy of I/R rats by measuring the ischemic area and neurological scores. The mechanisms of ginsenosides were also elucidated by immunohistochemistry and the western blot test. 

## 2. Results

### 2.1. Ginsenosides Attenuated CoCl_2_-Induced Cytotoxicity of PC12 Cells

**Figure 2:**
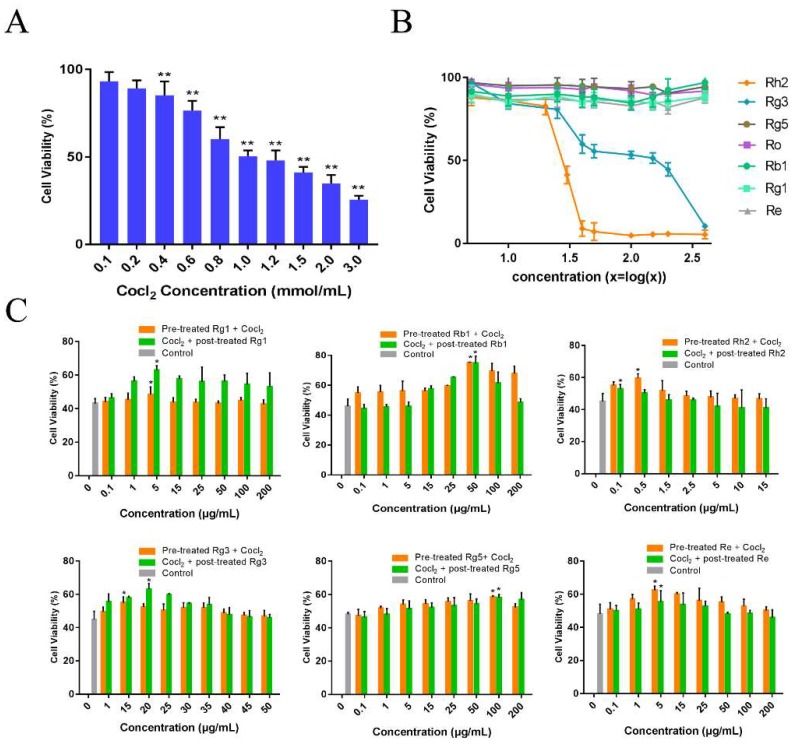
Protective effects of ginsenosides in CoCl_2_ injured PC12 cells. (**A**) Effect of CoCl_2_ on the cell viability of PC12 cells. The cells were induced with different concentration of CoCl_2_ for 24 h. Each value represents the mean ± SD, from six independent experiments (*n* = 6, ** mean *p* < 0.01 vs. control group); (**B**) Effect of ginsenoside Rg1, Rb1, Rh2, Rg3, Rg5 and Re (5, 10, 25, 40, 50, 100, 150, 200, 400 μg/mL) on PC12 cells cytotoxicity (*n* = 6). (**C**) The viability of PC12 cells pretreated with various concentrations of ginsenosides for 1 h before induced by CoCl_2_ or treated with ginsenosides after induced by CoCl_2_ for 1 h. The data are expressed as means ± SD (*n* = 6). * *p* < 0.05, compared with the control.

Firstly, the injury effects of CoCl_2_ at different concentrations on PC12 cells were measured using 3-[4,5-dimethyl-2-thiazolyl]-2,5-diphenyl-2H-tetrazolium bromide assay (MTT assay, Figure 2A), and we found that the survival rates of PC12 cells were inversely correlated with the CoCl_2_ concentration. When the concentration of CoCl_2_ was 1 mmol/L, the viability of PC12 cells was observed to be 50.4 ± 3.4%. Therefore, we chose 1 mmol/L CoCl_2_ in the following experiments. We further examined the effect of ginsenosides on CoCl_2_-induced PC12 cells injury. Through cytotoxicity experiments, when the concentrations of ginsenoside Rg1, Rb1, Rg5, Re were less than 400 μg/mL, the concentration of Rh2 was less than 20.77 μg/mL and the concentration of Rg3 was less than 97.9 μg/mL, the viability of PC12 cells was greater than 50%. Therefore, we chose these concentrations for pharmacodynamic experiments in vivo (Figure 2B). The protective effect of ginsenoside Rg1, Rb1, Rh2, Rg3, Rg5 and Re in CoCl_2_ injured PC12 cells was studied. PC12 cells were treated with ginsenosides at series concentrations: Rg1 (0.1 μg/mL to 200 μg/mL), Rb1 (0.1 μg/mL to 200 μg/ml), Rh2 (0.1 μg/mL to 15 μg/mL), Rg3 (1 μg/mL to 50 μg/mL), Rg5 (0.1 μg/mL to 200 μg/mL) and Re (0.1 μg/mL to 200 μg/mL) for 1 h before or after induced with CoCl_2_, respectively. As indicated in Figure 2C, the viabilities of PC12 cells increased to 62.99 ± 2.56% (*p* < 0.01) at 1 μg/mL for Rg1, 75.1 ± 0.37% (*p* < 0.01) at 50 μg/mL for Rb1, 59.47 ± 2.85% (*p* < 0.05) at 0.5 μg/mL for Rh2, 63.02 ± 3.56% (*p* < 0.01) at 20 μg/mL for Rg3, 58.37 ± 2.88% (*p* < 0.05) at 100 μg/mL for Rg5 and 62.35 ± 2.58% (*p* < 0.01) at 5 μg/mL for Re, which were the best of each group, respectively. Interestingly, ginsenoside Rg1 and Rg3 had better therapeutic effect on injured cells than preventive effect, while ginsenoside Rb1, Rh2, Rg5 and Re had better preventive effect.

### 2.2. Ginsenosides Reduced Neuronal Apoptosis, ROS Level and Increased Mitochondrial Membrane Potential of PC12 Cells

**Figure 3:**
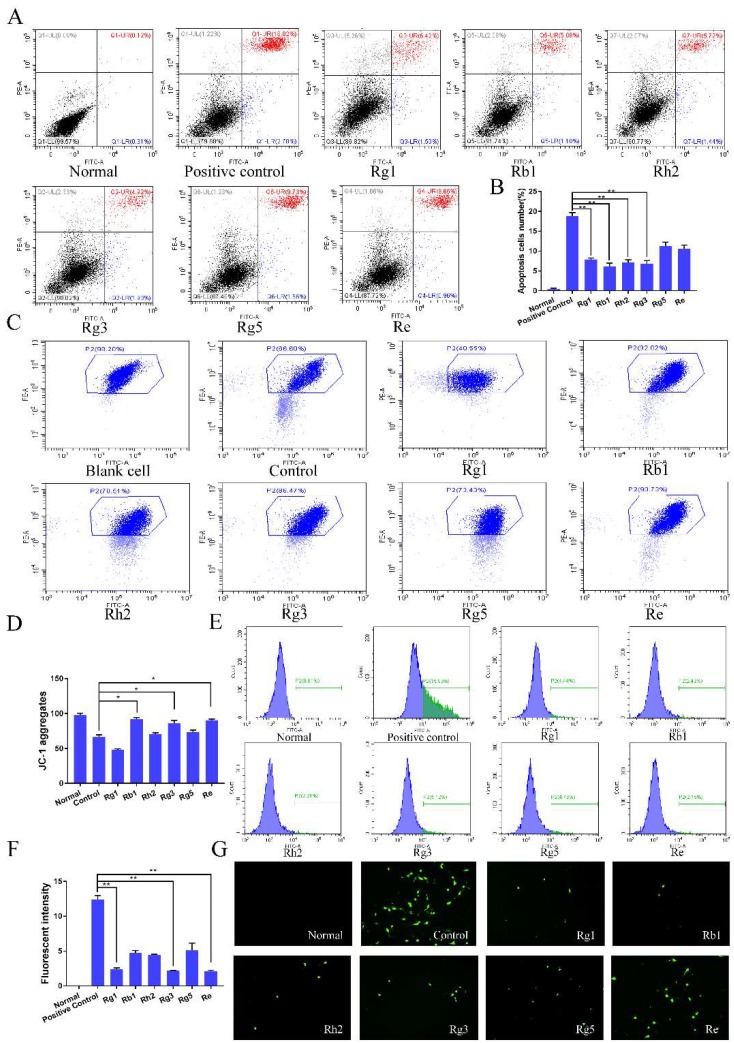
Effect of the ginsenosides on the apoptosis of PC12 cells. (**A**) Representative scatter plots of annexin V/PI analysis, (**B**) Percentage of apoptotic cells, including early and late apoptotic. (**C**) Representative dot plots of the distribution of JC-1 aggregates and JC-1 monomer of mitochondrial transmembrane potential (MMP). (**D**) The column chart of distribution of JC-1 cells percentage. (**E**) ROS level of PC12 cells measured by 2′,7′-dichlorofluorescin diacetate (DCFH-DA) dye dilution method. (**F**) The column chart of ROS level percentage. (**G**) Fluorescence intensity detected by inverted fluorescence microscope. Data were expressed as mean ± SD, *n* = 6. * *p* < 0.05, ** *p* < 0.01, compared with positive control.

The anti-apoptotic effects of ginsenosides were investigated using an Annexin V-FITC/PI assay through CoCl_2_-induced PC12 cells injury model. Compared with the apoptosis rate of 18.80% for the control group, the apoptosis rates of PC12 cells for ginsenoside Rg1, Rb1, Rh2 and Rg3 were reduced to 7.92% ± 0.33%, 6.18 ± 0.8%, 7.16 ± 0.66% and 6.85 ± 0.78% (*p* < 0.01) respectively (Figure 3A). The mitochondrial membrane potential reflects the integrity of mitochondrial function. When JC-1 stained cells are transformed from aggregate to monomer, it indicates mitochondrial dysfunction. As shown in Figure 3B, the MMPs of ginsenoside Rb1 group, Rg3 group and Re group were much higher than that of other groups. Quantitative results (Figure 3C) showed that compared with the control group, the mitochondrial function of the cells was effectively protected (*p* < 0.05) after the treatment of ginsenoside Rb1, Rg3 group and Re. As shown in Figure 3D, the ginsenosides groups reduced the level of ROS by approximately 80%, 82%, 82 % compared to the positive control group when treated with ginsenoside Rg1, Rg3, Re, respectively. 

### 2.3. Ginsenosides Attenuated both the Cerebral Ischemic Injury and the Neurological Deficits Caused by I/S

**Figure 4:**
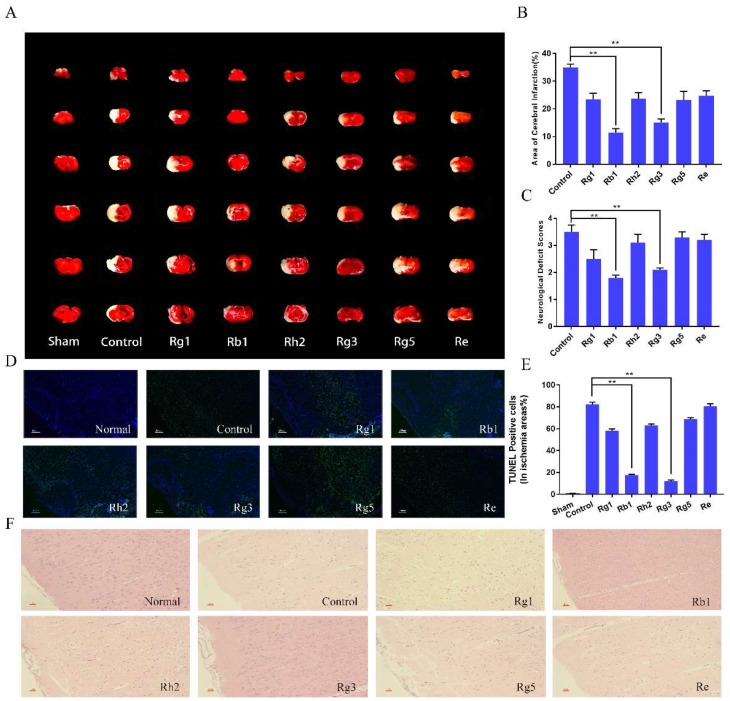
Effects of ginsenoside Rg1, Rb1, Rh2, Rg3, Rg5 and Re on infarct volume (**A**,**B**) and neurological functional outcome (**C**). Cerebral infarct volume was determined by 2,3,5-triphenyltetrazolium chloride (TTC) staining. In Fig. 4A, the red area represented normal brain tissue and the white area represented infarct area. Data were expressed as mean ± SD, *n* = 6. ** *p* < 0.01, compared with positive control. Representative pictures of TdT-mediated dUTP nick-end labeling (TUNEL) (**D**) and H&E staining (**F**) at 24 h after drug treatment and TUNEL positive cells quantitative analysis (**E**). Apoptosis was observed by TUNEL staining and original magnification was 100×. The blue indicated normal neuronal cells, and the green indicated apoptotic cells (200 μm). The damaged nerve cells in H&E staining were shown to be brown with a magnification of 200×. Data were expressed as mean ± SD, *n* = 6. ** *p* < 0.01.

To investigate the neuroprotective effects of ginsenosides in cerebral ischemia, middle cerebral artery occlusion (MCAO) in the rat model was established, which is a commonly used I/R model. We measured the infarct volume of I/R rats using 2,3,5-triphenyltetrazolium chloride (TTC) staining. Brain slices were prepared 24 h after MCAO and reperfusion. Compared with the control group, ginsenosides pretreatment groups could significantly reduce the infarct volume of I/R rats (Figure 4A,B). Ginsenoside Rb1 and ginsenoside Rg3 decreased the infarct volume to 67% and 57% of that of the vehicle treated group, respectively (*n* = 6, *p* < 0.01). Correspondingly, as shown in Figure 4C, ginsenoside Rb1 and Rg3 significantly improved the neurological functional recovery of the rats compared with the control group at 24 h after I/S (*n* = 6, *p* < 0.05).

The neuroprotective effect of ginsenosides was further investigated using TdT-mediated dUTP nick-end labeling (TUNEL) and Hematoxylin-eosin (H&E) staining. TUNEL staining was used to evaluate apoptosis, in which the apoptotic cells showed green fluorescence while normal neuronal cells showed blue fluorescence. As observed in Figure 4D, compared with the control group, ginsenoside Rb1 and Rg3 groups significantly reduced the green fluorescence intensity (*p* < 0.01), and the apoptosis rate was reduced to 19.3 ± 1.2%, 17.2 ± 1.4% by the two ginsenosides, respectively (*p* < 0.01, Figure 4E). The H&E staining was used to observe nerve cell damage in brain slices. As shown in Figure 4F, IS caused significant neuronal damage. In the control group, the neurons were loosely aligned, the nuclear boundaries of some neurons were unclear and some neurons exhibited a shrinking appearance. Ginsenosides could alleviate the neuronal damage to varying degrees, and ginsenoside Rb1and Rg3 showed the best protective effect.

### 2.4. Ginsenosides Reduced the Level of TNF-a, IL-6, and IL-1β in Penumbra Tissue of I/R Rats

**Figure 5:**
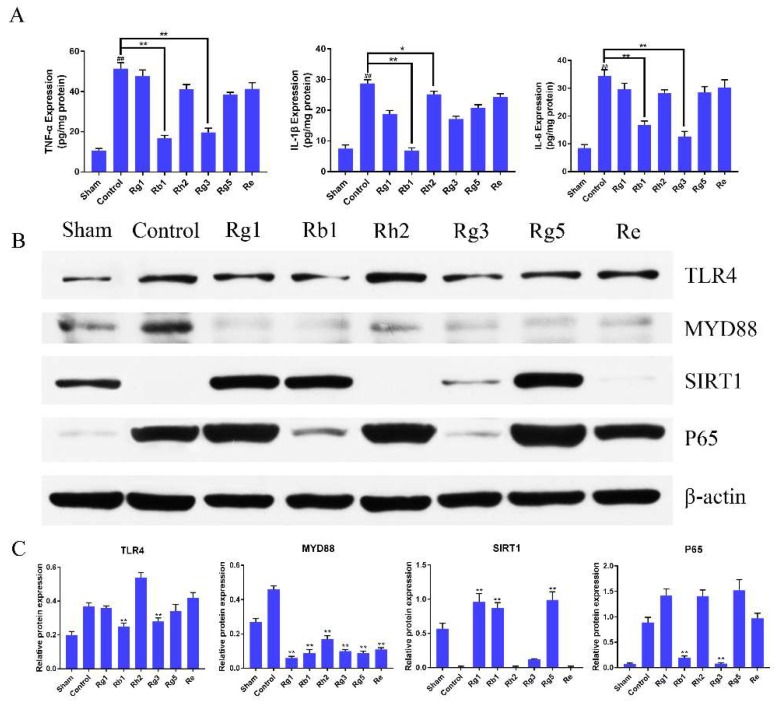
(**A**) Effects of ginsenosides treatment on the expression of TNF-a, IL-6, and IL-1β levels. Compared with the control group, the protein expression of ginsenoside Rb1 and Rg3 groups was significantly reduced. Values were expressed as means ± SD, *n* = 6; * *p* < 0.05 and ** *p* < 0.01 vs. the control group. (**B**,**C**) Effects of ginsenosides on the protein levels of TLR4, MyD88, SIRT1 and P65 in the penumbra after 1.5 h middle cerebral artery occlusion (MCAO) and 24 h reperfusion injury. Data were presented as mean ± SD. (** *p* < 0.01 vs. control group).

Inflammation and oxidative stress-related molecules lay a critical role in the pathophysiology of I/R injury. To elucidate the mechanisms of the ginsenosides, the inhibitory effect of ginsenosides on I/R-related expression of pro-inflammatory molecules was evaluated. The protein expression level of TNF-α, IL-6, IL-1β in the penumbra was measured using ELISA. It could be found from Figure 5A that the expression levels of TNF-a, IL-6, and IL-1β were significantly reduced in penumbra of ginsenoside Rb1 and Rg3 treated rats compared with those with vehicle after 1.5 h of ischemia and 24 h of reperfusion (*p* < 0.01), while the effect of other ginsenosides was not as strong as Rb1 and Rg3.

### 2.5. Ginsenosides Inhibited TLR4/MyD88 and SIRT1 Activation Signaling Pathway in Penumbra Tissue

The expression of TLR4, MyD88, SIRT1 and P65 in the penumbra was measured using western blot analysis. As seen in Figure 5B,C, the expression of TLR4 in ginsenoside Rb1 and Rg3 groups were sharply lower than those in other groups (*p* < 0.01). The expression of MyD88 in the control group was obviously higher than those in other groups (*p* < 0.01). The expression of SIRT1 in ginsenoside Rg1, Rb1 and Rg5 groups were much higher than those in other groups (*p* < 0.01). The expression of P65 in ginsenoside Rb1 and Rg3 groups were sharply lower than those in other groups (*p* < 0.01). Although the effects of six kinds of ginsenosides on protein expression were different, they all reduced the protein expression of the TLR4/MyD88 signaling pathway. In general, the effect on SIRT1 activation of ginsenoside Rb1 and the inhibition effect of TLR4/MyD88 protein expression of ginsenoside Rb1 and Rg3 were significantly stronger than that of other groups (*p* < 0.01). The results indicated that ginsenosides could effectively counteract brain damage by inhibiting TLR4/MyD88, regulating innate and adaptive immune inflammatory signaling and SIRT1 activation pathway. 

## 3. Discussion

In this study, six ginsenosides were selected to evaluate their effect on cerebral ischemia. CoCl_2_-induced PC12 cells were used as an in vitro model of hypoxia injury, and the protective effects of the ginsenosides on I/R-induced neuronal cell damage were compared by apoptosis, mitochondrial membrane potential and ROS content. The rat MCAO model was conducted to evaluate the in vivo efficacy by measuring the cerebral ischemic area and neurological score [18,19,20]. The results showed that ginsenoside Rb1 and Rg3 could significantly improve the neurological score and reduce the area of MCAO cerebral infarction. Ginsenoside Rb1 and Rg3 significantly reduced PC12 cell apoptosis and intracellular ROS content, and improved the protective effect of mitochondrial membrane potential in vitro. Meanwhile, the protective effect of other four ginsenosides on cerebral ischemic injury was not significantly different from that of the control group. Ginsenosides are structurally divided into ginseng dio- type and ginseng triol-type, and ginsenoside Rb1, Rh2, Rg3 and Rg5 belong to ginseng diol-type [13]. However, compared with ginsenoside Rb1 and Rg3, ginsenoside Rh2 and Rg5 had much less protective effect on cerebral ischemic injury, indicating that the efficacy of ginsenosides on I/R injury is dependent to their specific molecular structure.

In the pathophysiological process of I/R, in addition to hypoxia and energy failure, neuronal apoptosis, oxidative stress, excitatory amino acid increase and inflammation occurs as well [6,21,22]. The inflammatory response is accompanied by the entire process of I/R and considered a key part of the cascade of I/R pathogenesis [23,24]. In the early stages of cerebral ischemia, inflammation involves the activation of microglia and astrocytes, and a large number of adhesion molecules attach to the vascular endothelium, then cytokines and chemokines pass through the activated vessel wall to the ischemic site [7,25,26,27,28]. In the advanced stage, neuronal cells produce inflammatory cytokines, especially TNF-α, IL-1β and IL-6, which are caused by necrosis, apoptosis and autophagy, and lead to neuronal cell death [29,30]. 

**Figure 6:**
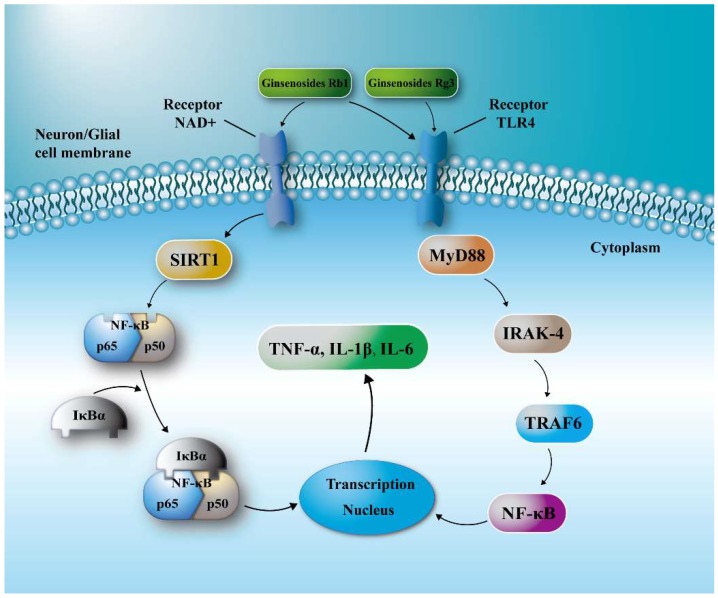
Schematic diagram for the neuroprotective effects of ginsenosides in I/R injury. Ginsenosides suppressed TLR4/MyD88 signaling and/or SIRT1 activation to reach a protective effect after I/R injury.

TLR4 is a congenital and adaptive immune cell receptor of the pathogen recognition receptor family [31,32]. MyD88 is a linker protein containing a TLR domain, a downstream signaling factor of the TLR signaling pathway, and a carboxy terminus that binds to the Toll-like receptor homology domain. And the C-terminal Toll region is similar to the cytoplasmic region of the IL-1β receptor. The death domain binds to the death domain of the IL-1β receptor-associated kinase and causes autophosphorylation of IRAK, followed by a series of activation reactions [33,34,35]. Eventually, the activation of I-κB is caused, which leads to the activation and translocation of nuclear factor-κB (NF-κB), the synthesis and release of inflammatory cytokines, and thus induces the inflammatory process of the body itself. SIRT1 can deacetylate the NF-κB subunit ReuA/i65, and the expression of genes involved in the regulation of NF-κB activity is deleted, which can reduce the binding of NF-κB to nuclear inflammatory genes, thereby reducing TNF-α, IL- release of 1β and IL-6 inflammatory factors [36,37,38]. Our study showed that ginsenosides can inhibit the expression of TLR4 and MyD88 proteins in the penumbra, and activate SIRT1 to down-regulate the expression of NF-κB, thereby reducing the release of inflammatory factors TNF-α, IL-1β and IL-6 (Figure 6). Among them, the effect of ginsenoside Rb1 and Rg3 was the strongest, therefore, they could significantly reduce both the cerebral ischemic injury and the neurological deficits of I/S rats, and has potential to be developed as an effective drug for cerebral ischemia.

## 4. Materials and Methods

### 4.1. Materials

Ginsenoside Rg1, Rb1, Rh2, Rg3, Rg5 and Re (purity > 98.0%), cobalt chloride (CoCl_2_), hydrogen peroxide and 2′,7′-dichlorofluorescin diacetate (DCFH-DA) were purchased from Dalian Meilun Biotech Co., Ltd. (Dalian, China). PC12 cells were purchased from the Cell Bank at the Chinese Academy of Sciences (Shanghai, China). 2,3,5-triphenyltetrazolium chloride (TTC) and 3-[4,5-dimethyl-2-thiazolyl]-2,5- diphenyl-2H-tetrazolium bromide (MTT) were provided by Sigma-Aldrich Company (St. Louis, MO, USA). 5,5-Dimethyl-1-pyrroline N-oxide was provided by Neobio Company (Shanghai, China). Rat MCAO model thread plug was purchased from Guangzhou Jialing Biotechnology Co., Ltd. (Guangzhou, China). Reactive Oxygen (ROS) Detection Kit and Apoptosis Kit were purchased from Shanghai Biyuntian Biotechnology Co., Ltd. (Shanghai, China). Hematoxylin-eosin (H&E) staining kits, interleukin-1β (IL-1β), interleukin-6 (IL-6) and tumor necrosis factor (TNF-α) ELISA kits were purchased from Nanjing Jiancheng Institute of Biotechnology (Nanjing, China). Rabbit anti-TLR4, rabbit anti-MyD88 rabbit anti-SIRT1 and rabbit anti-NF-κB were obtained from Proteintech, Inc. (Wuhan, China). Propidium iodide (PI) and annexin V-FITC were purchased from BioVision Inc. (Milpitas, CA, USA). Adult male Sprague-Dawley rats (250–300 g) were purchased from Sino-British BK Lab. Animal Co., Ltd. (Shanghai, China). The animal experiment protocol was approved by the Animal Experiment Ethical Committee of Fudan University (2018-10-YJ-WJX-01), and the in vivo experiments have been conducted in accordance with relevant national legislation on the use of animals for research.

### 4.2. Cytotoxicity of Ginsenosides

The cytotoxicity and the optimal concentrations of ginsenosides to PC12 cells were determined by MTT assay. PC12 cells were cultured in dulbecco’s modified eagle medium (DMEM) medium containing 10% fetal calf serum, 5% non-essential amino acids and 1% penicillin-streptomycin in an incubator with the atmosphere of 5% CO_2_ at 37 °C [39]. 4 × 10^2^ PC12 cells per well were seeded in 96-well plates. Ginsenoside Rg1, Rb1, Rh2, Rg3, Rg5 and Re were prepared as stock solution with dimethyl sulfoxide (DMSO) and diluted to the final concentrations with serum-free DMEM. The final concentration of DMSO was less than 0.1%. When PC12 cells had grown to anastomose, they were treated with ginsenosides of different concentrations (5, 10, 25, 40, 50, 100, 150, 200 and 400 μg/mL) for 24 h. Then, 50 μL MTT (2 mg/mL) was added into each well and incubated at 37 °C for 4 h. After that, the supernatant medium was removed and 150 μL DMSO was added to dissolve the formazan crystals. The optical density (OD, corresponding to absorbance) was measured with microplate reader at 570 nm, and the cell viability (%) was calculated with the following equation: cell viability (%) =$\frac{\mathrm{OD}\;\left(\mathrm{measured}\;\mathrm{value}\right)\;-\;\mathrm{OD}\;\left(\mathrm{blank}\;\mathrm{value}\right)}{\mathrm{OD}\;\left(\mathrm{control}\;\mathrm{value}\right)\;-\;\mathrm{OD}\;\left(\mathrm{blank}\;\mathrm{value}\right)}$ × 100%.

### 4.3. Effect of Ginsenosides on PC12 Cells Injury Induced by CoCl_2_

The effects of various ginsenosides on the apoptosis, mitochondrial transmembrane potential (MMP) and the level of reactive oxygen species (ROS) of PC12 cells injured by CoCl_2_ were studied to evaluate their therapeutic efficacy in vitro.

The injured cell model was established by adding CoCl_2_ to PC12 cells [40]. CoCl_2_ was dissolved and diluted to the final concentrations with serum-free DMEM. 3 × 10^3^ PC12 cells/well were seeded in 96-well plates and then CoCl_2_ solutions (0.1, 0.2, 0.4, 0.6, 0.8, 1.0, 1.2, 1.5, 2.0 and 3.0 mmol/mL) was added and incubated for 24 h.

To compare the preventive and therapeutic effects of ginsenosides, the cells were divided into three groups [41,42]: A. prevention group, in which the cells were pretreated with ginsenosides for 1 h before exposed to 1 mmol/mL CoCl_2_ for 24 h; B. therapy group, to which the ginsenoside was given 1 h after the administration of CoCl_2_, and incubated for another 23 h; C. control group, in which the cells were treated only with CoCl_2_ for 24 h.

The dose and the time of administration of each ginsenoside were determined according to the results in previous studies. PC12 cells were pretreated with ginsenoside Rb1 (50 μg/mL), ginsenoside Rh2 (0.5 μg/mL), ginsenoside Rg5 (100 μg/mL), ginsenoside Re (5 μg/mL) before adding 20 μL CoCl_2_ (1 mmol/L), or given ginsenoside Rg1 (5 μg/mL), ginsenoside Rg3 (20 μg/mL) after adding CoCl_2_, respectively. The final concentration of CoCl_2_ was 1 mmol/L and the cells were induced for 24 h. Afterwards, the supernatant was replaced with serum-free DMEM. 

The apoptosis of PC12 cells was measured using the Annexin V-FITC/PI double staining kit [43]. The 2′,7′-dichlorofluorescin diacetate dilution (10 μM) or Annexin-V (1 mg/mL) and PI (1 mg/mL) were added and incubated for 15 min in the dark at room temperature. The cells were quantitatively analyzed by flow cytometry, the proportion of early apoptotic cells (Annexin V+, PI-) and late apoptotic cells (Annexin V-, PI+) in the cell population was obtained, and the fluorescence intensity was quantified with flow cytometry at 488 nm or 525 nm.

Mitochondrial function was evaluated by using a cyanine dye JC-1 to assess MMP. The fluorescence of JC-1 stained cells shifted from red (JC-1 aggregates) to green (JC-1 monomer) while MMP breakdown. So, the JC-1 aggregates represented the fluctuation of MMP. JC-1 solution (5 μg/mL) was added into PC12 cells and incubated for 30 min in the dark at 37 °C under the same condition. MMP of PC12 cells was detected by flow cytometry at 488 nm or 525 nm (FACS Calibur, BD, San Jose, CA, USA).

Intracellular ROS levels were determined with Reactive Oxygen Species Assay Kit. 2′,7′-dichlorofluorescin diacetate (DCFH-DA) dilution (10 μM) was added and incubated for 30 min at 37 °C. The cells were then resuspended with 300 μL PBS after washing twice with cold PBS. The apoptosis, ROS level of PC12 cells was detected by flow cytometry at 488 nm or 525 nm (FACS Calibur, BD, San Jose, CA, USA).

### 4.4. Establishment of Middle Cerebral Artery Occlusion (MCAO) Rat Model 

Middle cerebral artery occlusion (MCAO), a commonly used I/R model, was established to evaluate the pharmacodymics of ginsenosides [44]. After fasted for 12 h, SD rats (250 ± 10 g) were injected intraperitoneally with 10% chloral hydrate (4 mL/kg), and then fixed in supine position. The skin was cut on the neck along the line, and the submandibular gland was separated to expose neck vessels. The left common carotid artery (CCA), external carotid artery (ECA) and internal carotid artery (ICA) were unilaterally separated. After CCA and ECA were ligated, ICA was clamped and a small hole was cut on it. A monofilament nylon thread coated with poly-L-lysine was inserted into the ICA through the hole, and push forward about 18–19 mm to the starting point of the middle cerebral artery (MCA) to fix the nylon thread. After 1.5 h of ischemia, the nylon suture was removed to enforce reperfusion. Then, the skin was sutured and 1 mL of physiological saline was intraperitoneally injected. The body temperature of the rats was maintained at 37.5 °C ± 0.5 °C with a heating pad during the whole process of surgery and ischemia.

### 4.5. Effect of Ginsenosides on MCAO Rats

The rats were randomly divided into eight groups (*n* = 6), i.e., the sham group, MCAO + PBS as control group, MCAO + Rg1 group, MCAO + Rb1 group, MCAO + Rh2 group, MCAO + Rg3 group, MCAO + Rg5 group and MCAO + Re group. Two hours after reperfusion, different ginsenosides were completely dissolved in DMSO and diluted with PBS. The final concentration of DMSO was less than 2%, and 0.5 mL was injected from the tail vein to the rats, respectively. The dose of ginsenosides were 10 mg/kg. For the sham and the control groups, 0.5 mL PBS was given. Approximately 24 h later, the neurological deficit scores of the rats were measured, which were classified as following: 0: no motor disability (normal); 1: limbs weakness and cannot fully extend (mild); 2: circle to one side (moderate); 3: dump to one side (severe); 4: unconscious or unable to ambulate spontaneously (critical). After that, the rats were anesthetized with 10% chloral hydrate (i.p., 4 mL/kg) and sacrificed by perfusing the hearts with saline. The brains were removed quickly and frozen at −20 °C for 20 min. 2 mm coronal slices were cut using a rodent brain matrix. The sections were stained with 2% 2,3,5-triphenyltet-razolium chloride (TTC) monohydrate for 20 min at 37 °C, and then fixed with 4% formalin for 15 min. The stained slices were photographed with a camera (Olympus em10). The infarction area was calculated by subtracting the non-infarct area of the ipsilateral side from the area of the contralateral side using Image J software (National Institutes of Health, Bethesda, MD, USA). 

Neuronal apoptosis of the ischemia region was detected with the TUNEL kit following the instruction [45]. The percentage of apoptosis cells in the ischemic area was calculated as apoptosis cells/total cells in ischemic area × 100%. The brain tissue injury was observed by hematoxylin-eosin (H&E) staining [46]. The cerebral ischemia tissues were stained with H&E solution according to standard procedure [47]. The photographs (200 magnification) were observed under optical microscope.

### 4.6. Measurement of Cytokines by ELISA

Six rats of each group were sacrificed at 24 h after I/S. The brain homogenates (approximately 50 mg) were obtained from the ischemic hemisphere after centrifuging at 13,600× *g* for 5 min to remove cellular debris. The supernatants were stored at −80 °C until analysis. The inflammatory cytokines, including TNF-α, IL-1β and IL-6 in the supernatant were measured using commercial ELISA kits.

### 4.7. Western Blot Analysis

The proteins extracted from the cortex of the ischemia side and the corresponding areas of the sham group rats were analyzed by western blot [48]. The brain tissues were also homogenized with OMNI homogenizer (BioSpec Products, Inc., Bartlesville, OK, USA, level 10, 2 × 30 s) in lysis buffer (50 mmol/L Tris-HCl buffer, pH 7.4, containing 0.1 mol/L NaCl, 0.1% Triton X-100 and 1 tablet/10 mL). The lysates were incubated for 30 min on ice and centrifuged at 6950× *g* for 10 min. The proteins in this supernatant were measured using a BCA Protein Assay Kit (Beyotime, Shanghai, China). The proteins (50 μg) were separated on SDS-polyacrylamide gels and transferred onto a polyvinylidene fluoride (PVDF) membrane (Millipore Corporation, Billerica, MA, USA). The membrane was blocked with 5% nonfat dry milk in tris-buffered saline and then incubated with different primary antibodies (TLR4; MyD88; SIRT1 and NF-κB/p65) (1:1000 dilution) overnight at 4 °C and horseradish peroxidase conjugated antibody at room temperature for 1 h. The expression of proteins was tested by western blot with enhanced chemiluminescence (ECL) method and imaged using ChemiDoc XRS (BIO RAD, Hercules, CA, USA). Protein signals were quantified by scanning densitometry. The protein levels of pro-inflammatory mediators were expressed as relative integrated intensity normalized versus β-actin.

### 4.8. Statistical Analysis

All data were expressed as mean ± SD values. Graphpad Software (GraphPad Software Inc., La Jolla, CA, USA) for Windows was employed for statistical analysis and subjected to one-way analysis of variance (ANOVA) and one-way repeated measures ANOVA. The values from the control group and those from the ginsenosides groups were compared by using the Bonferroni *t* test with *p* < 0.05 considered statistically significant. 

## 5. Conclusions

We chose six ginsenosides, namely ginsenoside Rg1, Rb1, Rh2, Rg3, Rg5 and Re, which were reported to be effective for ischemia-reperfusion (I/R) treatment, to compare their protective effects on cerebral I/R injury. The results indicated that all six ginsenosides were effective in protecting the brain against ischemic injury, and ginsenoside Rb1 and Rg3 have the strongest therapeutic activities in all the tested ginsenosides. The neuroprotective mechanism of the ginsenosides is associated with TLR4/MyD88 and SIRT1 activation signaling pathways, and can reduce cerebral ischemic injury by inhibiting NF-κB transcriptional activity and the expression of proinflammatory cytokines, including interleukin-1β (IL-1β), tumor necrosis factor-α (TNF-α) and interleukin-6 (IL-6).
